# Exploring the HIF-1α signalling pathway and the mechanism of YiQiHuoXue decoction against Precancerous Lesions of Gastric Cancer based on Network Pharmacology and Molecular Docking

**DOI:** 10.7150/jca.95938

**Published:** 2024-05-11

**Authors:** Suting Qian, Feifei Xie, Haoyu Zhao, Xin Hua, Ting Jiang, Chuchu Zhang, Zepeng Cao, Jianshun Yu, Qingsheng Liu

**Affiliations:** Hangzhou Hospital of TCM affiliated to Zhejiang Chinese Medical University, Hangzhou, China.

**Keywords:** YiQiHuoXue Decoction, Precancerous Lesions of Gastric Cancer, HIF-1α signalling pathway, network pharmacology, experimental verification

## Abstract

Precancerous Lesions of Gastric Cancer (PLGC) are an essential step in the advancement of Gastric cancer (GC). Early intervention represents the most effective strategy to impede the development of PLGC. However, additional research is necessary to comprehend the molecular mechanism of PLGC. YQHXD is originated from Si Wu Decoction, has been utilized as an empirical formula for the treatment of PLGC for several years. In this study, we employed network pharmacology, molecular docking, and experimental validation to examine the inhibitory and ameliorative properties of YQHXD on PLGC. Multiple databases were utilized to gather genetic information on drugs in PLGC and YQHXD, in order to obtain cross-targets. We discovered 142 common targets between YQHXD and PLGC. GO and KEGG enrichment analyses indicate that YQHXD treatment of PLGC might be linked with cellular response to oxygen levels and the HIF-1α signaling pathway. Finally, we performed in vitro experiments, of which the results reveal that YQHXD mitigates gastric mucosal atrophy, intestinalization, and heterogeneous hyperplasia, and reduces the expression of inflammatory factors in rats. Therefore, we considered that YQHXD has the potential to delay the PLGC process by inhibiting the HIF-1α signaling pathway.

## Introduction

The detection rate of gastric cancer has increased annually, with over one million diagnoses each year. Despite the decline in mortality rates due to advances in modern medicine, gastric cancer remains the third leading cause of cancer-related deaths 1, 2. Gastroscopy was followed up in patients diagnosed with chronic atrophic gastritis, intestinal metaplasia, or dysplasia, and 1.4% of patients were diagnosed with high-grade adenoma/dysplasia or invasive neoplasia within 5 years 3. PLGC range from normal mucosa to chronic gastritis (chronic inflammation of the gastric mucosa), mucosal atrophy (loss of gastric glands), intestinal metaplasia, dysplasia, and cancer 4. To prevent and block the progression of gastric cancer from the stage of PLGC, it is important to detect it early. Patients with PLGC are typically asymptomatic and are often diagnosed through gastroscopy biopsy 5. A study had shown that the normal gastric mucosa is about 1/256, gastritis is 1/85, atrophic gastritis is 1/50, intestinal metaplasia is 1/39, and dysplasia is 1/19 in gastroscopy 6. Therefore, early diagnosis and treatment are the most effective ways to prevent and treat PLGC 7. However, Western medicine has limited treatment of PLGC. Traditional Chinese Medicine (TCM) has been proven to have certain advantages in the treatment of PLGC 8. Classical Chinese medicine formulas and some herbal chemical components have been used to treat PLGC, which can carry out multi-pathway and multi-target comprehensive intervention 9.

YQHXD is a protocol prescription for the treatment of chronic atrophic gastritis (CAG) accompanied by intestinal metaplasia/dysplasia, which has been applied clinically by my supervisor's research group for more than 10 years. It is composed of Chinese herbs such as Codonopsis, astragalus and panax notoginseng. The whole decoction has beneficial effects of Qi and blood circulation, removing blood stasis and relieving pain, and has good clinical effect. Previous animal experiments studies have shown that YQHXD can regulate the microenvironment homeostatic state of gastric mucosa cells, increase their antioxidant stress ability, improve hypoxia state, resist the stimulation and damage of cytokines and inflammatory factors on gastric mucosa, and slow or inhibit the process of PLGC. However, the exact mechanism of action of YQHXD remains to be further studied.

Network pharmacology is based on the rapid development of systems biology and computer technology. It is a new approach covering traditional pharmacology, bioinformatics, chemical informatics and network biology. Based on the interaction network of "drug - component - target gene - disease", network pharmacology can systematically observe the intervention and influence of drugs and their active components on the target gene of disease, so as to reveal the mechanism of the synergistic action of TCM compounds on the human body 10.

Through network pharmacological studies, it is predicted that YQHXD can regulate the HIF-1α signaling pathway to treat PLGC. YQHXD may inhibit or delay the HIF-1α signaling pathway by regulating the expression of HIF-1α, VEGF, iNOS gene and protein, which are key genes in the HIF-1α signaling pathway in gastric mucosa tissue, thus inhibiting angiogenesis and inflammatory reaction during PLGC process. Therefore, the mechanism of HIF-1α signaling pathway in PLGC and the intervention effect of YQHXD on PLGC in newborn rats induced by MNNG were investigated, which laid an experimental foundation for the further application of TCM in the clinical treatment of PLGC and provided a new idea for the later treatment of PLGC with TCM.

## Materials and methods

### Screening and target identification of the active chemical ingredients of YQHXD

**Figure 1:**
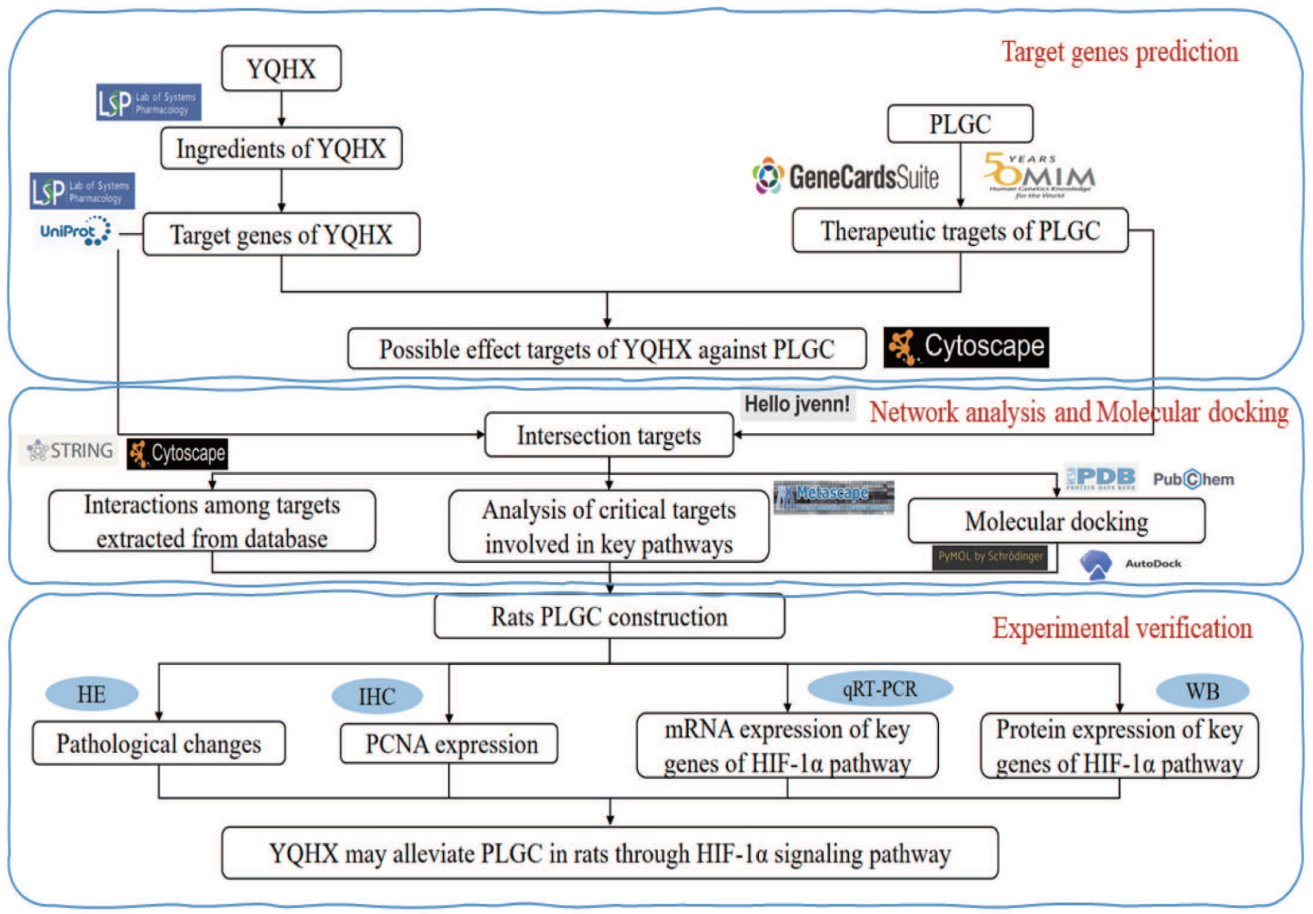
The technical route flow chart of the research.

All phytochemical constituents in the 8 herbs (Codonopsis Radix; Astragalus; Amomun Villosum; Finger Citron; Szechuan Lovage Rhizome; Pseudo-ginseng; Angelicae Sinensis Radix; Curcuma Zedoaria, the details were shown in Table 1) of YQHXD were screened from the TCM Systems Pharmacology Database and Analysis Platform (TCMSP, http://tcmspw.com/tcmsp.php), a pharmacological platform for Chinese herbal medicine systems to probe into the correlation between drugs, genes and diseases. Oral bioavailability (OB) ≥ 30% and drug-likeness (DL) ≥ 0.18 were used as screening criteria to search the active ingredients and matching target proteins of the above drugs. For uniform standards, all the target names were transformed to normative gene symbols using the UniProt database (https://www.uniprot.org/). The technical route is shown in Figure 1.

### PLGC-related target retrieval

“Precancerous Lesions of Gastric Cancer” was used as the keyword to search for related target genes in the Genecards (https://www.genecards.org/), Online Mendelian Inheritance in Man (OMIM) (https://www.omim.org/) and Drugbank (https://go.drugbank.com/) databases. The final list of PLGC-related targets were generated after removing false positives. Uniprot database was used to standardize the PLGC-related targets.

### Analyzing and building networks

In order to identify potential targets for YQHXD in treating PLGC, the active ingredients-related targets YQHXD of were mapped against PLGC-related targets. Using the online tool jvenn (http://jvenn.toulouse.inra.fr/app/example.html) to map relevant target connections. we constructed the "active component-action target" relationship network of YQHXD using Cytoscape 3.9.1 (https://cytoscape.org/index.html).

### Network construction for protein-protein interactions (PPIs)

The STRING database(https://string-db.org/) was used in order to construct the PPI network. A selection option of "Multiple Proteins", a protein species of "Homo sapiens", a medium confidence level of 0.700, and an interaction threshold of "hide disconnected notes" have been chosen for the interaction analysis. The default settings were used for the remaining parameters. The data were imported into Cytoscape 3.9.1 for visualization and construct a topological network of YQHXD-related and PLGC-related targets.

### Enrichment analysis and Pathway Analysis

Database for Metascape (https://metascape.org/) was used to analyze overlapping genes based on gene ontology (GO) function analysis and Kyoto Encyclopaedia of Genes and Genomes (KEGG) pathway enrichment analysis. GO encompasses three aspects of biology, namely biological processes (GO-BP), molecular functions (GO-MF), and cellular components (GO-CC). The KEGG pathway enrichment analysis was used to identify potential targets of YQHXD for treating PLGC. We identified significant functions and pathways using the critical value of P<0.05.

### Molecular docking

The 3D molecular structures (SDF format) of the active ingredients were downloaded from PubChem(http://pubchem.ncbi.nlm.nih.gov) and converted to mol2 using OpenBabel 3.1.1. RCSB Protein Data Bank (PDB) (https://www.rcsb.org/) was used to download the 3D structures of the core targets (PDB format). It was selected based on the following criteria: 1) it was a human protein; 2) it had a high resolution; and 3) it was the original protein. Water molecules and original ligands were removed from the core target protein using PyMOL 2.4.1. As well as hydrogenating the core target and converting it to pdbqt, the small molecule compound rotation bond was also set and saved as pdbqt using AutoDock 4.2.6 software. The results were visualized using PyMOL software using semi-flexible molecular docking to dock core target proteins and small molecules.

### Experiment

#### Experimental animals

36 healthy 4-5-week-old SPF-grade SD rats, purchased from Shanghai Slaughter Laboratory Animal Co., Ltd. (license number: SCXK (Shanghai) 2012-0002), were fed to 2 months in a 2:1 mating breeding, and healthy suckling rats born for 3 days were selected for subsequent experiments.

#### Experimental drugs

Tokyo Kasei Industry Co. provided N-methyl-N'-nitro-N-nitrosoguanidine (MNNG, Lot: ZZRRL-OR), prepared at a concentration of 800mg/L and stored at 4°C.

YQHXD comprises the following components: Codonopsis pilosula (Franch.) Nannf.; Astragalus aboriginum Spreng; Amomum villosum Lour; Citrus medica L.; Ligusticum striatum DC.; Panax notoginseng (Burkill) F.H. Chen; Angelica sinensis (Oliv.) Diels; Curcuma zedoaria (Christm.) Roscoe. The herbs were made into a fluid infusion by the Chinese medicine preparation laboratory of the First Hospital of Zhejiang Chinese Medical University.

#### Experimental Main Reagents

The main reagents for the experiment are shown in Table 2.

#### Animal grouping, model production, intervention, and issue sampling

The 3-day-old mammary rats were randomly selected to be given 0.1ml MNNG by gavage after regular point breastfeeding for 2 hours every day, and the same number of control rats were given 0.1ml normal saline. The gastric mucosal lesions were dynamically observed after 10 days of feeding, and the modeling was completed at about 24 weeks. After successful modeling, 32 rats were randomly selected to form the model group, high-dose group, medium-dose group and low-dose group, 8 rats each. 8 rats without modeling at the same time were taken as normal control group. The rats in the normal and model groups were given 0.01 ml/g of saline, 0.0384 g/g of YQHXD by gavage in the high-dose group, 0.0192 g/g of YQHXD by gavage in the middle-dose group, and 0.0096 g/g of YQHXD by gavage in the low-dose group. The rats were executed after 12 weeks of feeding. The whole stomach was removed and dissected along the large curved side of the stomach for histopathological observation. The remaining gastric tissues were subjected to immunohistochemistry for Proliferating Cell Nuclear Antigen (PCNA) expression, Quantitative real-time reverse transcription-polymerase chain reaction (qRT-PCR) and western blot (WB).

#### Gastric histopathology detected by HE staining

The gastric mucosa tissue was fixed with neutral formaldehyde for 24h, dehydrated by gradient ethanol, treated with xylene transparent, paraffin-embedded at 58°C after paraffin dipping, sliced at 5µm, baked at 60°C for Hematoxylin-eosin (HE) staining, microscopic observation of pathological changes in gastric mucosa tissue. The Chinese Consensus on Chronic Gastritis (2017, Shanghai) and the International Grading Standard of dysplastic lesions Pavoda^11^ were used as the corresponding diagnostic criteria for pathological changes of gastric mucosa.

#### Detection of proliferation of gastric mucosa cells by IHC

Envision two-step immunochemistry was used to detect PCNA expression in the gastric mucosa of each group of rats, and the gastric mucosal proliferation index was calculated.

#### Detection of mRNA expression of key genes of HIF-1α signaling pathway in rat gastric tissue by qRT-PCR

qRT-PCR was used to determine HIF-1α, VEGF, and iNOS mRNA levels. Total RNA was isolated from gastric tissue by a Total RNA Extraction Kit. The RNA purity was determined. The total RNA concentration was calculated, and the final concentration was adjusted to 0.25ng/µl. Subsequently, cDNA was prepared by reverse transcription kit, incubated at 37°C for 15min, and terminated at 85°C for 5sec. A total of 10µL of CHamQTM SYBRR qPCR Master Mix was used for the PCR reaction.

The cDNA was amplified using primers for 40 cycles at the following temperatures: 95°C for 3 minutes, 95°C for 5 seconds, and 60°C for 30 seconds of annealing. The products were analyzed with Ct values and β-actin was used as internal reference. 2-ΔΔCt indicated the relative expression levels of HIF-1α, VEGF, iNOS and β-actin mRNA. The primer sequences are presented in Table 3.

#### Detection of protein expression of key genes of HIF-1α signaling pathway in rat gastric tissue by WB

Samples were subjected to WB to detect the levels of HIF-1α, VEGF, and iNOS. Total proteins were extracted with the Total Protein Extraction Kit. The protein concentration was determined with the BCA Protein Assay Kit, and the protein concentration was adjusted to 10μg/μl. Appropriate amount (60 µg) of protein was loaded on 10% sodium dodecyl sulfate-polyacrylamide gel electrophoresis (SDS-PAGE) and transferred to polyvinylidene difluoride (PVDF) membranes. The membranes were sealed with 5% skim milk powder and incubated at 4°C overnight with primary antibodies (HIF-1α 1:1000 dilution; VEGF 1:1000 dilution; iNOS 1:500 dilution; GAPDH 1 : 1000 dilution). The membranes were washed with TBST 3 times for 10 min each time and incubated with secondary antibodies for 1h at room temperature. The film was placed in the ECL luminous developer and the double infrared imaging system to detect the automatic exposure. The gray value of the film was analyzed by Image Studio Ver 5.2 software, and the gray value of the sample protein/GAPDH gray value was used as the relative expression value for comparison.

#### Statistical analysis

The data were expressed 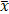
±s. Using SPSS25.0 software, a comparative analysis was performed using One-Way Analysis of Variance (ANOVA) to compare among multi-groups followed by the least significant difference (LSD) comparison between any two groups. Non-parametric rank sum test was used for counting data. The difference in the test level of bilateral a ¼ 0.05 and p < 0.05 was considered statistically significant.

## Results

### Active compounds and targets of YQHXD

**Figure 2:**
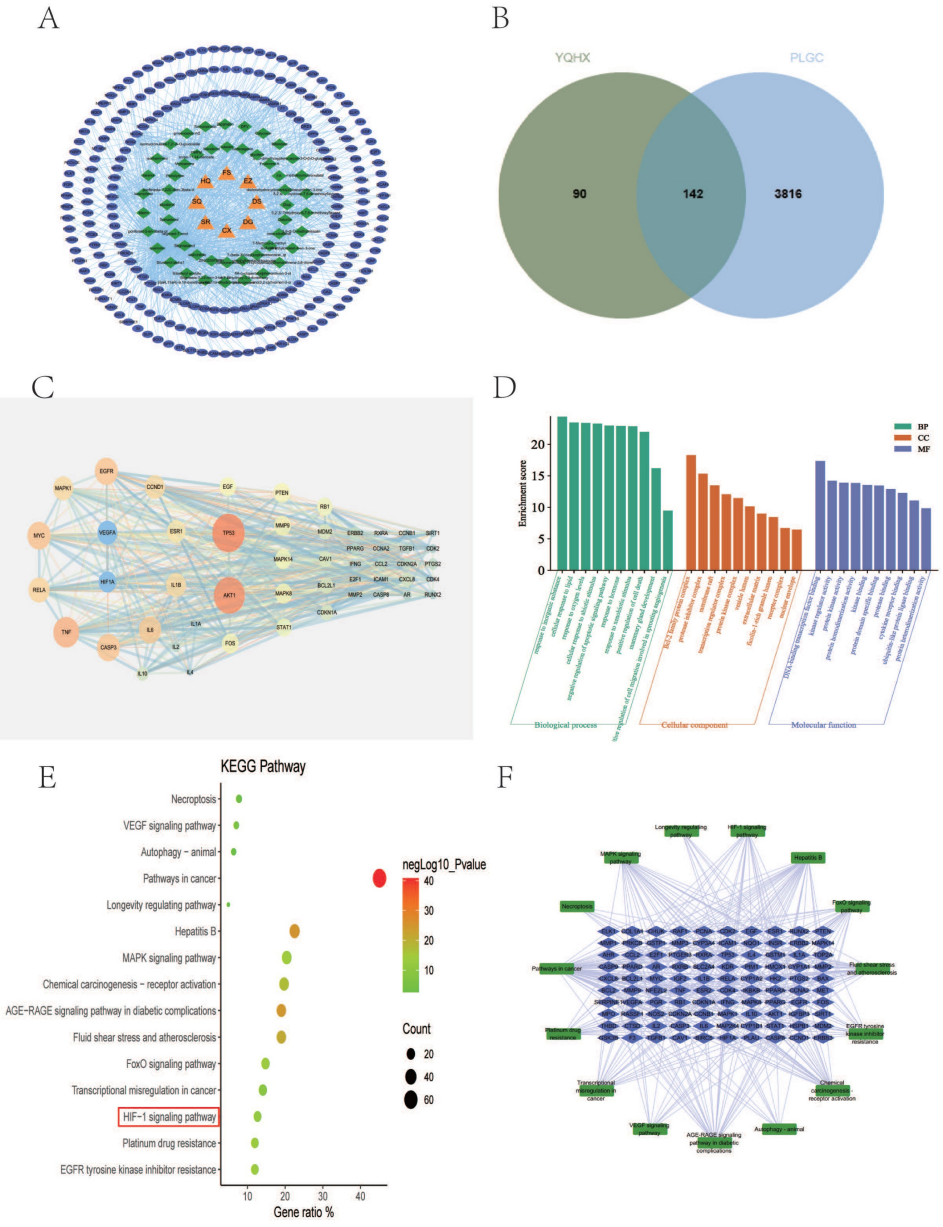
Network pharmacology analysis for YQHXD in the treatment of PLGC. (A) targets Venn diagram of YQHXD against PLGC. (B) herbs-components-disease-targets network. (C) PPI of the common targets related to PLGC interacting with YQHXD. (D) GO analysis. (E) KEGG pathway analysis. (F) target-pathway network of YQHXD on treating PLGC.

Using the TCMSP database, retrieved the active compounds and effective target information for each herb in YQHXD, and converted to a uniform protein name by Uniprot database. There are 8 herbs in YQHXD, including 101 target in Codonopsis Radix (Dangshen), 201 in Astragalus (Huangqi), 47 in Amomun Villosum (Sharen), 22 in Finger Citron (Foshou), 26 in Szechuan Lovage Rhizome (Chuanxiong), 177 in Pseudo-ginseng (Sanqi), 47 in Angelicae Sinensis Radix (Danggui), 21 in Curcuma Zedoaria (Ezhu). A total of 232 targets were obtained from YQHXD after recovering and weighting (Fig. 2A). The number of active ingredients and related targets of YQHXD are shown in Table 4.

### Potential YQHXD targets for treating PLGC

A total of 3958 gene targets associated with PLGC were obtained via the GeneCards and OMIM databases after removing reduplicative entries. A Venn diagram was established through Jvenn(http://jvenn.toulouse.inra.fr/), and 142 active ingredients of YQHXD were associated as potential targets in PLGC treatment (Fig. 2B).

### Key nodes in the PPI network

To explore the core pharmacological mechanism of YQHXD in the treatment of PLGC, we constructed a PPI network using the top 50 overlapping genes. Among them, the top 15 bases are TP53, AKT1, TNF, MYC, CASP3, EGFR, IL6, RELA, VEGFA, IL1B, CCND1, MAPK1, ESR1, HIF-1α, FOS (Fig. 2C).

### Enrichment analysis of the targets of YQHXD in the treatment of PLGC

GO enrichment and KEGG pathway enrichment analysis were performed for 142 common targets using metascape database. We found the biological process function was mainly related to response to inorganic substance, cellular response to lipid and response to oxygen levels. The cellular component was primarily associated with Bcl-2 family protein complex、protease inhibitor complex、membrane raft. The molecular function was found mainly related to DNA-binding transcription factor blinding, kinase regulator activity, protein kinase activity (Fig. 2D). KEGG analysis results showed that the treatment of PLGC with YQHXD was mainly related to oxidative stress and inflammatory response. The ralated 10 pathways were Pathways in cancer, Hepatitis B, MAPK signaling pathway, Chemical carcinogenesis-receptor activation, AGE-RAGE signaling pathway in diabetic complications, Fluid shear stress and atherosclerosis, FoxO signaling pathway, Transcriptional misregulation in cancer, HIF-1 signaling pathway, Platinum drug resistance, EGFR tyrosine kinase inhibitor resistance (Fig. 2E-F).

### Molecular docking in YQHXD and HIF-1a signaling pathway

To further explore the regulatory effect of YQHXD active compounds on the regulation of the HIF-1a signaling pathway on target proteins, we selected Stigmasterol, beta-sitosterol, Perlolyrine, Diop, methylicosa-11,14-dienoate, hederagenin, FA, quercetin and Mandenol active ingredients as ligands, and HIF-1a, VEGF and NOS targets as receptors for molecular docking simulation. The information was shown in Supplementary Table S1.

Results showed that the above 6 active compounds were able to spontaneously bind to the 3 targets naturally, ligands without 3D structure were excluded (Diop, methylicosa-11,14-dienoate, Mandenol). The conformation of the compound binding to the target is more stable the lower the binding energy 12.

**Figure 3:**
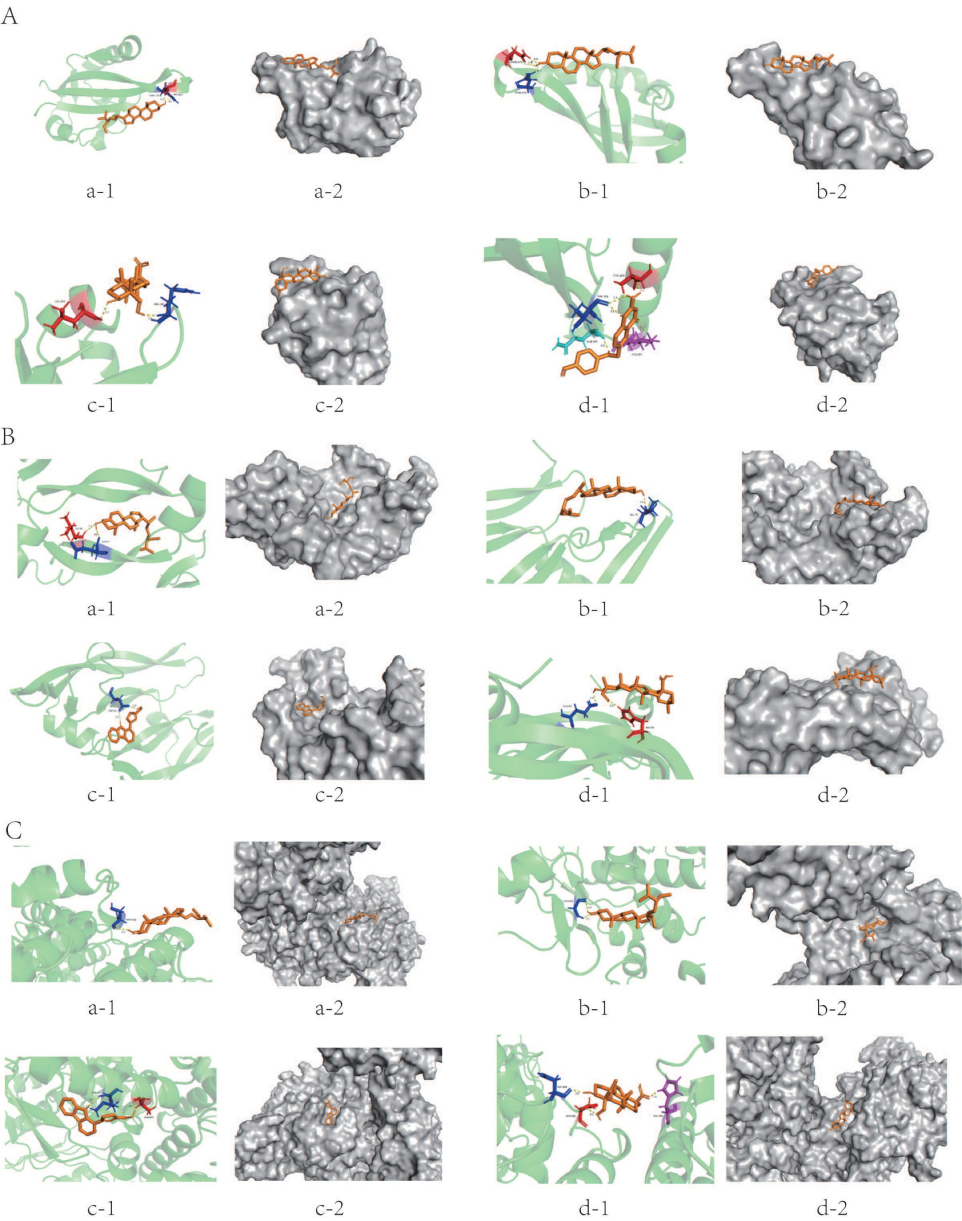
Molecular docking model diagram: (A) HIF-1a: (a) HIF-1a-Stigmasterol; (b) HIF-1a-Beta-sitosterol; (c) HIF-1a-Hederagenin; (d) HIF-1a-FA; (B) VEGF: (a) VEGF-Stigmasterol; (b) VEGF-Beta-sitosterol; (c) VEGF-Perlolyrine; (d) VEGF-Hederagenin; (C) NOS: (a) NOS-Stigmasterol; (b) NOS-Beta-sitosterol; (c) NOS-Perlolyrine; (d) NOS-Hederagenin; (a) Molecular docking model, the orange represents the 3D structure of the component ligand, the green represents the 3D structure of the target receptor, and the other colors represents the amino acid residues; (b) In the enlarged structure of the docking site, the yellow represents the hydrogen bond formed between the ligand and the amino acid residue, and the number represents the bond length; (c) Combination diagram of the surface structure of the target receptor and the component ligand, the orange represents the 3D structure of the ligand, and the gray represents the receptor surface structure.

Except for Perlolyrine and quercetin, the absolute value of the binding energy of all ligands and HIF-1a receptors were less than 5 KJ/mol. Except for FA and quercetin, the absolute value of the binding energy of all ligands and VEGF and NOS receptors were lower than 5 KJ/mol. The compounds had high binding energy to the targets, which is an indication that their docking results were good (Table 5). Stigmasterol was connected to the amino acid residues ASN-326 and THR-327 of HIF-1a through two hydrogen bonds (bond length are 1.9 Å and 2.3 Å) to form a relatively stable conformation (binding energy = -6.29kJ/ mol, Figure 3A-a); Stigmasterol also formed 2 hydrogen bonds with amino acid residues LEU-59 and GLU-57 of VEGFA (bond lengths are 2.2 Å, and 2.7 Å, respectively) for stable binding (binding energy = -6.72kJ/ mol, Figure 3B-a).FA stably bound to THR-290, THR-301, GLN-299 and LYS-297 amino acid residues of HIF-1a by forming 5 hydrogen bonds (bond lengths are 1.9Å , 2.0Å , 2.1Å , 2.1Å and 2.4 Å, respectively) (binding energy = -5.03 KJ/mol, Figure 3A-d);Hederagenin formed 3 hydrogen bonds with the amino acid residues ASP-384, ASP-386, and HIS-408 of NOS(the bond lengths were 1.9 Å, 2.0 Å, and 2.2 Å, respectively), these resulted in a more stable docking (binding energy = -6.95kJ/ mol, Figure 3C-d).

The main molecular docking model diagram is presented in Figure 3.

### Gastric tissue pathological changes in rats

The pathological changes of gastric mucosa epithelium were observed after HE staining.

The lamina propria of normal gastric mucosa epithelial cells were densely arranged, regular in shape and complete in structure. The nuclei were round or oval, and there was no evidence of overt atypia or infiltration by inflammatory cells.

In the model group, a gradual increase in intracellular inflammatory cells, such as lymphocytes and plasma cells, in the stomach lining, and the intrinsic glands were atrophied or even reduced, occasionally cystic dilatation was observed, accompanied by different degrees of dysplasia.

**Figure 4:**
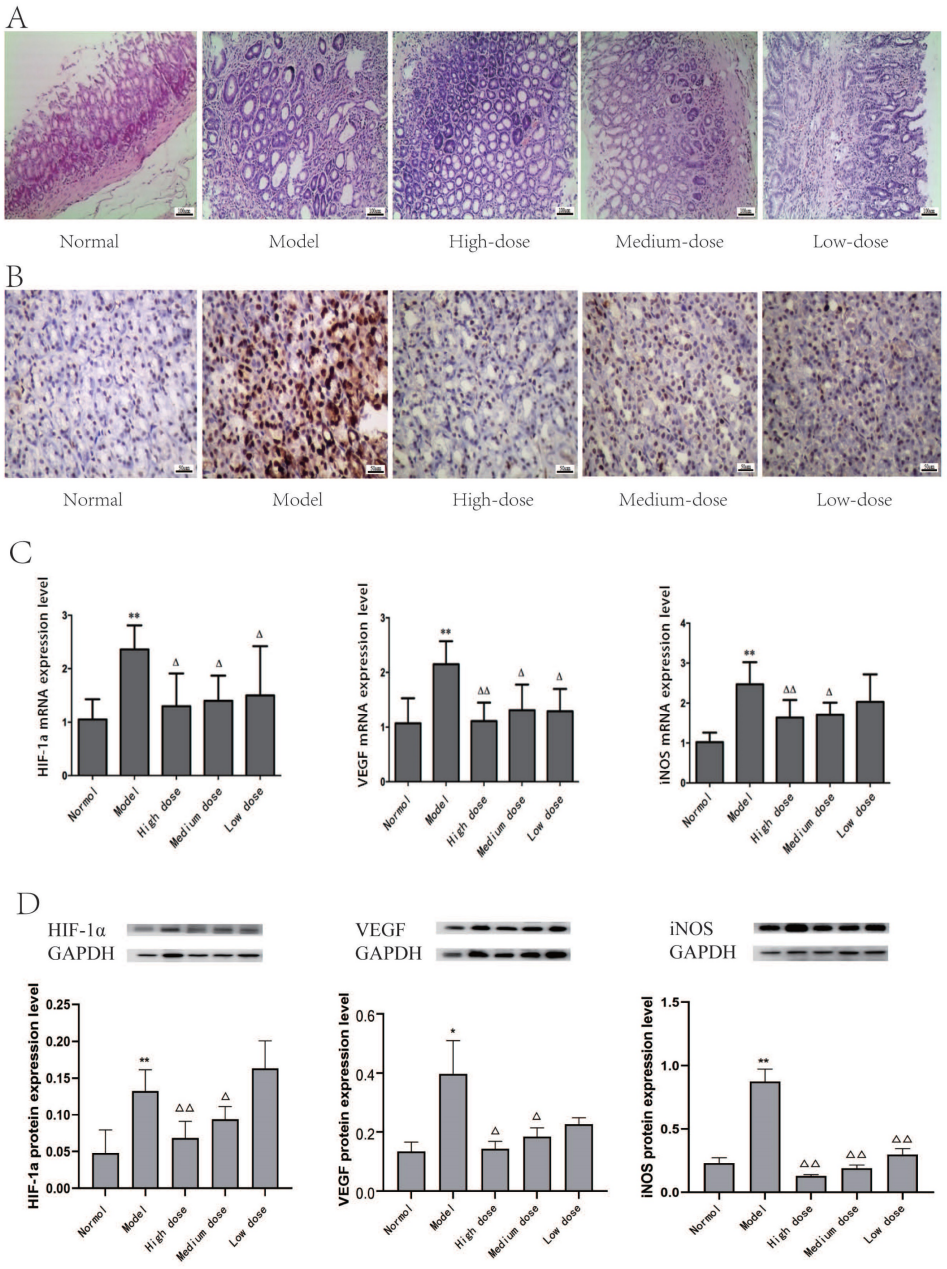
The experimental results of YQHXD in the treatment of PLGC rats. (A) HE stained pathological section of gastric tissue (HE *100). (B) Expression of PCNA in gastric mucosa of YQHXD (IHC *400). (C) mRNA expression of key genes of HIF-1α signaling pathway in gastric mucosa of rats. (D) Protein expression of key genes of HIF-1α signaling pathway in gastric mucosa of rats. (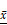
±s, n=8; Compared with normal group *P<0.05, **P<0.01, compared with model group Δ*P*<0.05, ΔΔ*P*<0.01).

In YQHXD group, a small amount of inflammatory infiltration of neutrophils, lymphocytes and monocytes was observed in stomach mucosa, and there has been a reduction in the degree of atrophy. There was no severe inflammation, atrophy and dysplasia of gastric mucosa in high-dose group (Fig. 4A).

### The PCNA expression in rat gastric tissue

The color and distribution of gastric mucosa nuclei was uniform in normal group. In the model group, the gastric mucosa nucleus was positively expressed, and the microscopically observed yellowish brown, brownish yellow or brownish fine granules with disordered distribution and uneven depth. With the increase of drug dose, the positive staining of gastric mucosa in YQHXD group was lightened and the disorder was reduced (Fig. 4B).

### mRNA expression of key genes of HIF-1α pathway PLGC in rat gastric tissue

Compared with the normal group, mRNA of HIF-1α, VEGF and iNOS were significantly expressed in model group *(P<0.05, P<0.01)*. The expressions of HIF-1α, VEGF and iNOS mRNA in gastric mucosa of rats were lower than those in model group *(P<0.05)*, and showed a downward trend with the increase of dose after using YQHXD (Fig. 4C).

### Protein expression of key genes of HIF-1α pathway PLGC in rat gastric tissue

The expressions of HIF-1α, VEGF and iNOS proteins in the gastric mucosa HIF-1α signaling pathway in model group were significantly higher than those in normal group *(P<0.05, P<0.01)*.

The expression of HIF-1α, VEGF and iNOS protein in gastric mucosa of rats in the medium-high dose group was decreased compared with that in the model group *(P<0.05)*, the expression of HIF-1α and iNOS protein in the high-dose group decreased the most* (P<0.05, P<0.01)* (Fig. 4D).

## Discussion

GC is a highly lethal malignant tumor with a poor prognosis worldwide, and its incidence is increasing in China 13. The process of GC involves the malignant transformation of CAG into GC, which is referred to as PLGC. This stage is crucial in slowing, blocking, and reversing the development of gastric malignancy 14. The current chemotherapeutic agents and treatment regimens for gastric cancer at this stage have limited therapeutic potential, high side effects, and poor prognosis, resulting in low 5-year and even 10-year survival rates in clinical practice. Therefore, there is an urgent need to improve these existing treatment modalities 15.

PLGC is a pathological concept proposed in recent years, and its symptoms are variable and not fixed. Clinically, it is often defined as " Stomach ruffian" in Chinese medicine based on its symptoms such as stuffiness, fullness or vague pain in the stomach and epigastrium, often accompanied by belching and acid reflux, loose stools or nausea and vomiting, and the consensus discussed by experts at the Fifth National Academic Exchange Conference on Spleen and Stomach Diseases 16. Through the experience of ancient and modern medical doctors such as " Jin Gui Yao Lue " and "PiWei Lun ", "weakness" is the essence and "stasis" is the core of the disease. The pathogenesis of the disease is mainly a mixture of deficiency and actuality, the original deficiency and the standard actuality. The location of the disease is mainly about the spleen and stomach, and the key lies in the weakness of qi in the spleen and stomach 17, 18. Currently, there are numerous chemical compositions available for classical TCM formulas and herbal isolates used in the treatment of PLGC. These compositions can be utilized for multi-pathway and multi-targeted comprehensive interventions, resulting in remarkable outcomes 19.

The YQHXD is an agreed prescription for the treatment of CAG with Intestinal Metaplasi (IM)/Dysplasia (Dys) applied by the Department of Gastroenterology of our hospital for more than ten years, consisting of Codonopsis Radix, Astragalus, Amomun Villosum, Finger Citron, Szechuan Lovage Rhizome, Pseudo-ginseng, Angelicae Sinensis Radix, Curcuma Zedoaria. Water-soluble polysaccharides isolated from Codonopsis Radix induce apoptosis of MKN45 cells through upregulation of Bax/Bcl-2 ratio and activation of caspase-3 20. Astragalus can reduce the angiogenesis of tumor and the invasive ability of gastric cancer cells, and can accelerate the process of apoptosis by high expression of Bcl-2 and low expression of Bax, partially inhibiting chromatin condensation and fragmentation 21. Volatile oil of A. villosum (VOAV), total flavonoids of A. villosum (FNAV) and other residues of A. villosum (RFAV) in Amomun Villosum can contribute to rapid apoptosis of MFC cells by regulating ROS-mediated mitochondrial pathway, promoting increased endogenous ROS and mitochondrial membrane potential collapse 22. Finger Citron inhibits tumor cell proliferation and invasion and induces apoptosis by downregulating Cyc-D1, MMP and bcl-2 through siRNA-mediated Zeb1 silencing replication to promote Zeb1 overexpression 23. Ligustrazine in Szechuan Lovage Rhizome may improve microcirculatory disorders and reduce damage to the gastric mucosa by increasing gastric blood flow and reducing inflammatory mediators 24. Total saponins of panax can reduce the expression of Vemintin, SNAIL, MMP2 and MMP9 and increase the expression of P21, Caspase-3 and Bax in gastric cancer cell lines, thus promoting apoptosis of tumor cells and hindering their proliferation, migration and invasion 25. The alkali-soluble polysaccharides contained in Angelica have the activity of stimulating the proliferation of immune cells (splenocytes, abdominal macrophages and natural killer cells), as well as promoting the release of cytokines (TNF-α, IL-2) and gamma interferon (IFN-γ) to promote apoptosis of tumor cells. Curcuma longa extract exhibits potent anti-cancer, anti-oxidative stress and anti-inflammatory activities by modulating relevant signaling pathways such as Nrf2, NF-κB and histone modified epigenetic/epigenomic pathways 26.

Based on network pharmacology, 232 potential gene targets of YQHXD were screened. 142 co-acting gene targets were obtained after Intersected with 3958 PLGC disease-related targets. Enrichment analysis identified 738 biological processes, 73 cellular components, 128 molecular functions, and 165 signalling pathways associated with the co-acting gene targets. We found that YQHXD mainly regulates TP53, AKT1, TNF, MYC, CASP3, EGFR, IL6, RELA, VEGFA, IL1B, CCND1, MAPK1, ESR1, HIF-1α, FOS, and other related target genes. Therefore, we speculate that YQHXD may be involved in regulating hypoxia and oxidative stress response, and treating PLGC through HIF-1α or other related signaling pathways.

To investigate the potential molecular mechanism of YQHXD for treating PLGC. The study is based on the results of network pharmacology, including the screening of active ingredients of the compound and the targets in the PPI network. Six compounds, stigmasterol, beta-sitosterol, perlolyrine, hederagenin, FA, and quercetin, were screened for molecular docking with key genes on the HIF-1a signaling pathway, HIF-1α, VEGF, and NOS proteins. The results of network pharmacology were confirmed through molecular docking. The docking results indicate that each protein forms a stable complex with at least four compounds. It was demonstrated that YQHXD could act on PLGC through the HIF-1α pathway.

HIF signaling pathway is a key signaling pathway that affects biological growth and development, regulates cell proliferation, differentiation and apoptosis, and repairs chronic inflammatory damage in tissues, and plays an important regulatory role in gastric cancer, breast cancer, liver cancer, colorectal cancer and other cancers 27, 28. HIF-1 mainly contains two subunits, HIF-1α and HIF-1β. HIF-1α is usually induced by hypoxia 29. The expression of HIF-1α is mainly related to the oxygen concentration in the body, and it is easy to be excited under low oxygen conditions, so it is often studied as a hypoxic index 30. At present, it has been found that there are multiple target genes in the HIF-1α pathway, such as vascular endothelial growth factor (VEGF), inducible nitric oxide synthase(iNOS), lactate dehydrogenase-A (LDHA), erythropoietin (EPO), connective tissue growth factor (CTGF), endothelin (ET), etc. These target genes contain hypoxia response element (HRE). When the oxygen concentration of the body is low, the HIF-1α binding site in the target gene HRE is opened. HIF-1α binds to promote its transcription, and the cell then produces a series of regulatory measures for the hypoxic environment to maintain homeostasis 31. Through network pharmacology studies, we consider that YQHXD may treat PLGC by regulating target genes such as HIF-1α, VEGF, and related inflammatory factors in the HIF-1α signaling pathway.

HIF-1α regulates the expression of multiple target genes in the body, including VEGF. As one of the most important cytokines in cancer cells inducing neovascularization, VEGF plays an important role in the occurrence, evolution and metastasis of cancer cells 32, 33. VEGF is susceptible to various factors, such as ischemia, hypoxia, cytokines, tumor suppressor genes, etc. Therefore, VEGF is one of the most important regulatory genes for HIF-1α 34. Studies have shown a strong relationship between the activation of HIF-1α expression and the inflammatory response. Inflammation has been shown to be one of the important causes of cancer 35. iNOS is an important enzyme that mediates inflammatory processes, and abnormal upregulation of iNOS is associated with certain types of cancer as well as the pathophysiology of inflammatory diseases 36. Under inflammatory reaction, inflammatory cells and epithelial cells release Reactive Oxygen Species (ROS) and Reactive Nitrogen Species (RNS), resulting in DNA damage, which induces a highly mobile group 1 inflammatory microenvironment characterized by hypoxia. Hypoxia induces HIF-1α and iNOS, thereby increasing levels of RNS and ROS within cells, leading to DNA damage and poor prognosis 37. Therefore, HIF-1α, VEGF and iNOS are important regulators in PLGC, and the activation of HIF-1α signaling pathway is closely related to the occurrence and development of PLGC.

In this experiment, compared with the normal group, the positive expression of PCNA in the gastric mucosa of rats in the model group increased significantly, and the expression of HIF-1α, VEGF, iNOS mRNA and protein also increased significantly, confirming that it was accompanied by the activation of HIF-1α signaling pathway during PLGC. After YQHXD treatment, atrophy, intestinal chemistry and dysplasia were reduced to different degrees in each group, and IOD and PCNA expression were reduced compared with the model group, indicating that YQHXD played a reverse inhibitory role in the carcinogenesis process of gastric mucosal tissue, which could slow down the process of gastric mucosal carcinogenesis. At the same time, the results of this study found that the expression of HIF-1α, VEGF, iNOS mRNA and protein decreased with the increase of dose compared with the high-dose group, medium-dose group and low-dose group, indicating that the high-dose group had the best therapeutic effect. Through this study, it is found that in the process of MNNG-mediated PLGC in rats, HIF-1α, VEGF and iNOS in the HIF-1α signaling pathway are closely related to the PLGC process, which can be used as one of the detection indicators for the early diagnosis of PLGC, which provides a preliminary experimental basis for targeted therapy PLGC, and accumulates relevant experience for better clinical application. It also proves that YQHXD can be used to slow down the process of PLGC in the early clinical stage, and at the same time, it can improve the prognosis of patients and improve their living standards when combined with western drug therapy or targeted drug therapy in the later stage.

The active ingredients, targets and side effects are an important aspect of the modern research of TCM. TCM has the characteristics of multi-component, target and signaling pathway in tumor treatment. We explore their possible relevant targets and pathways through network pharmacology, and verify their correlation through experiments. In this study, the important targets and signaling pathways of YQHXD were screened by network pharmacology and verified by animal experiments to explore the mechanism and effect of PLGC, which provided a scientific basis and a basis for the clinical use of YQHXD. However, the composition of traditional Chinese medicines is more complex, and the exact active ingredients of YQHXD still lack research support, and it is impossible to determine whether there is an interaction between related pathways, which will be the next important research content.

## Conclusions

Through network pharmacology analysis and animal experiments, it can be basically determined that YQHXD can inhibit or delay the progression of PLGC by downregulating the key target genes HIF-1α, VEGF and iNOS in the HIF-1α pathway, which provides a scientific basis for the clinical use. In this study, rats were used as experimental subjects, so clinical validation through multi-center clinical trials was still required.

## Supplementary Material

Supplementary figure and table.

## Figures and Tables

**Figure 5:**
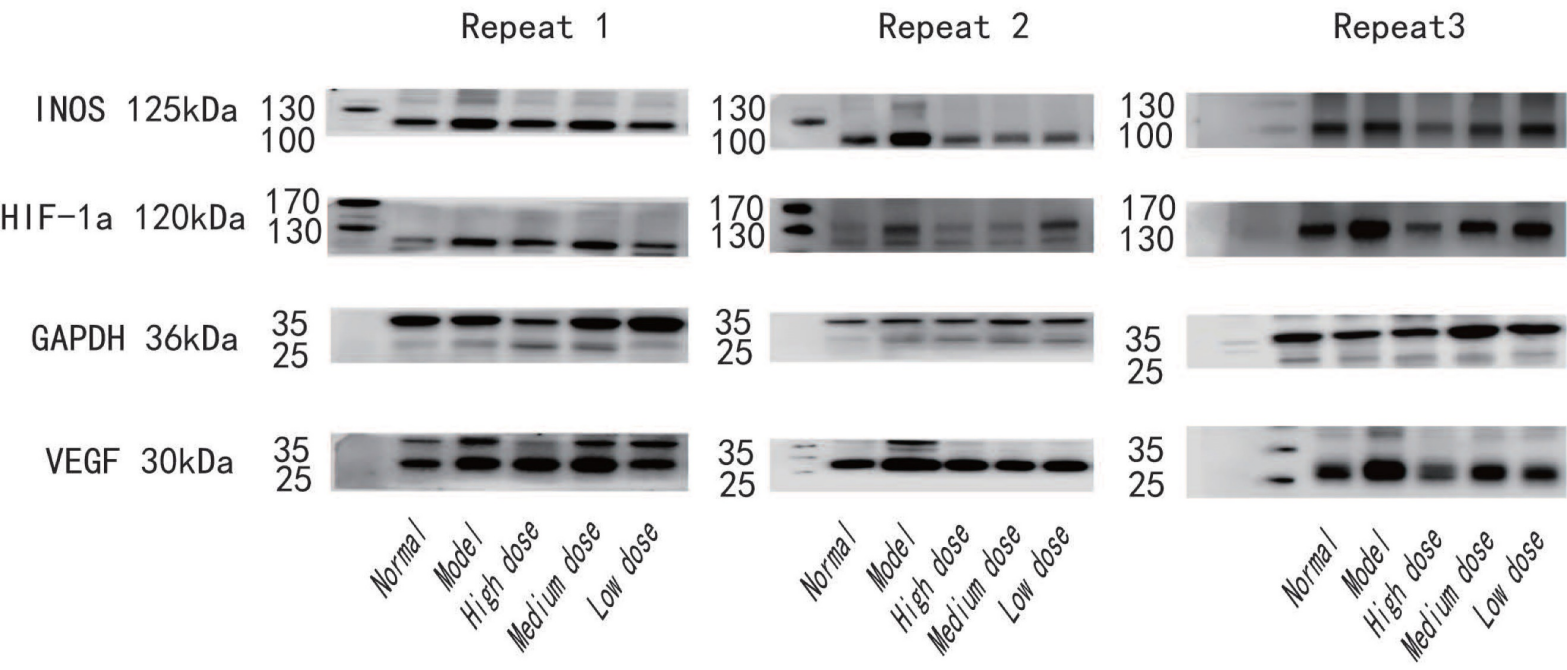
Example of original western blot for three repeats. Shows the whole blot after cutting membrane at molecular weight for INOS (125kDa), HIF-1a (120kDa), GAPDH (36kDa) and VEGF (30kDa).

**Table 1:** Full botanical plant names

English names	Latin names
Codonopsis Radix	*Codonopsis pilosula* (Franch.) Nannf.
Astragalus	*Astragalus aboriginum* Spreng
Amomun Villosum	*Amomum villosum* Lour
Finger Citron	*Citrus medica* L.
Szechuan Lovage Rhizome	*Ligusticum striatum* DC.
Pseudo-ginseng	*Panax notoginseng* (Burkill) F.H.Chen
Angelicae Sinensis Radix	*Angelica sinensis* (Oliv.) Diels
Curcuma Zedoaria	*Curcuma zedoaria* (Christm.) Roscoe

The plant name has been checked with http://www.theplantlist.org.

**Table 2:** Experiment reagents

Reagent name	Production company	Article or batch number
PCNA rabbit monoclonal antibody	CST USA, Inc.	Lot: 0004
HIF-1α (D2u3T) rabbit monoclonal antibody	CST USA, Inc.	Lot: 0001
VEGF mouse monoclonal antibody	NOVUS Inc.	Lot: NB100-664
iNOS mouse monoclonal antibody	R&D Inc.	Lot: CRF0819031
GAPDH mouse monoclonal antibody	Proteintech Inc.	Lot: 10013030
Goat anti-mouse IgG (H+L)	Hangzhou Unitech Biotechnology Co.	Lot: A00413
Goat anti-rabbit IgG (H+L)	Hangzhou Unitech Biotechnology Co.	Lot: A00613
Protein Ladder (10-250KD)	Hangzhou Unitech Biotechnology Co.	Lot: A00751

**Table 3:** Primer sequence table

Primer	Sequence		Product Size
HIF-1α	sense primer	5'-GAAATGGCCCAGTGAGAAAG-3'	116bp
	anti-sense primer	5'-CTTCCACGTTGCTGACTTGA-3'	
VEGF	sense primer anti-sense primer	5'-GCACGTTGGCTCACTTCCAG-3' 5'-TGGTCGGAACCAGAATCTTTATCTC-3'	107bp
iNOS	sense primer anti-sense primer	5'-GCTACACTTCCAACGCAACA-3' 5'-CATGGTGAACACGTTCTTGG-3'	116bp
β-actin	sense primer anti-sense primer	5'-TGTTGCCCTAGACTTCGAGCA-3' 5'-CCATACCCAGGAAGGAAGGCT-3'	155bp

**Table 4:** The number of active ingredients and related targets of YQHXD.

Chinese medicine	Chinese name	Latin names	Active ingredient	Target
Codonopsis Radix	Dangshen (DS)	*Codonopsis pilosula* (Franch.) Nannf.	21	101
Astragalus	Huangqi (HQ)	*Astragalus aboriginum* Spreng	20	201
Amomun Villosum	Sharen (SR)	*Amomum villosum* Lour	10	47
Finger Citron	Foshou (FS)	*Citrus medica* L.	5	22
Szechuan Lovage Rhizome	Chuanxiong (CX)	*Ligusticum striatum* DC.	7	26
Pseudo-ginseng	Sanqi (SQ)	*Panax notoginseng* (Burkill) F.H.Chen	8	177
Angelicae Sinensis Radix	Danggui (DG)	*Angelica sinensis* (Oliv.) Diels	2	47
Curcuma Zedoaria	Ezhu (EZ)	*Curcuma zedoaria* (Christm.) Roscoe	3	21

**Table 5:** The binding energy of key active ingredients docked with target molecules.

Target protein	Active ingredient	Pubchem CID	Amino acid residue	Binding energy (KJ/mol)
HIF-1a	Stigmasterol	5280794	ASN-326 THR-327	-6.29
	beta-sitosterol	222284	ASN-329 ASN-326	-5.59
	Perlolyrine	160179	GLU-257 ASP-256	-4.82
	hederagenin	73299	LEU-262 ARG-311	-5.65
	FA	6037	THR-290 THR-301 GLN-299 LYS-297	-5.03
	quercetin	5280343	LYS-297 GLN-299 THR-301 THR-290	-3.75
VEGF	Stigmasterol	5280794	LEU-59 GLU-57	-6.72
	beta-sitosterol	222284	ILE-70	-5.35
	Perlolyrine	160179	ASP-61	-5.14
	hederagenin	73299	GLU-57 HIS-54	-5.68
	FA	6037	GLU-57	-3.92
	quercetin	5280343	GLU-57 GLY-86	-4.54
NOS	Stigmasterol	5280794	SER-392	-6.38
	beta-sitosterol	222284	GLY263	-6.15
	Perlolyrine	160179	MET-428 ALA-425	-5.26
	hederagenin	73299	ASP-384 HIS-408 ASP-386	-6.95
	FA	6037	GLU-75 PRO-370	-3.57
	quercetin	5280343	ASN-374 GLU-377	-3.41

## Abbreviations

PLGC: Precancerous Lesions of Gastric Cancer
HE: Hematoxylin-Eosin
IHC: Immunohistochemistry
WB: Western Blotting
GC: gastric cancer
CAG: chronic atrophic gastritis
MNNG: N-methyl-N'-nitro-N-nitrosoguanidine
VEGF: vascular endothelial growth factor
iNOS: inducible nitric oxide synthase
LDHA: lactate dehydrogenase-A
EPO: erythropoietin
CTGF: connective tissue growth factor
ET: endothelin
HRE: hypoxia response element
ROS: Reactive Oxygen Species
RNS: Reactive Nitrogen Species
