# Supplementary material for: Exploring the HIF-1α signalling pathway and the mechanism of YiQiHuoXue decoction against Precancerous Lesions of Gastric Cancer based on Network Pharmacology and Molecular Docking
Source: J Cancer. 2024 May 11;15(11):3566–79. doi: 10.7150/jca.95938 (PMC11134427; doi:10.7150/jca.95938)
Supplement: Supplementary file 1 — Supplementary figure and table. [file jcav15p3566s1.pdf]

Supplementary

Table S1. Detailed information of shared active ingredients derived from YQHXD.

Mol ID/CAS	Mol name	Mol structure	Mol formula	OB/GIA	BBB	DL	Related Chinese medicine
MOL000449	Stigmasterol	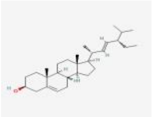	$C_{29}H_{48}O$	43.83	1	0.76	DS SR SQ DG
MOL000358	beta-sitosterol	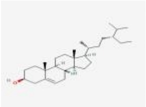	$C_{29}H_{50}O$	36.91	0.99	0.75	SR SQ DG
MOL002140	Perlolyrine	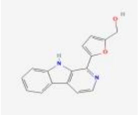	$C_{16}H_{12}N_2O_2$	65.95	0.15	0.27	DS CX
MOL000296	hederagenin	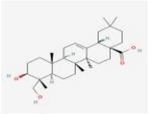	$C_{30}H_{48}O_4$	36.91	0.96	0.75	HQ EZ
MOL000433	FA	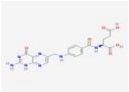	$C_{19}H_{19}N_7O_6$	68.96	-2.59	0.71	HQ CX
MOL000098	quercetin	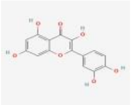	$C_{15}H_{10}O_7$	46.43	-0.77	0.28	HQ SQ

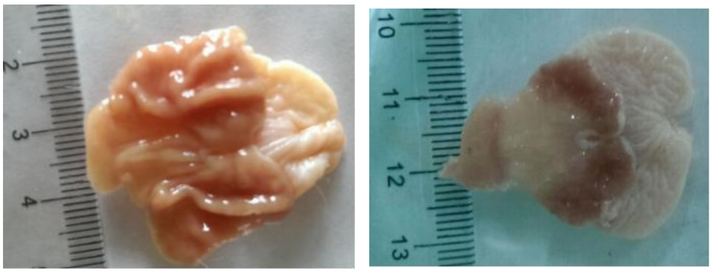

Normol Model

Figure S1 The images of gastric mucosa of PLGC rats in normal and model group.
